# A Population Genomics Perspective on the Emergence and Adaptation of New Plant Pathogens in Agro-Ecosystems

**DOI:** 10.1371/journal.ppat.1002893

**Published:** 2012-09-27

**Authors:** Eva H. Stukenbrock, Thomas Bataillon

**Affiliations:** 1 Max Planck Institute for Terrestrial Microbiology, Marburg, Germany; 2 Bioinformatics Research Center, Aarhus University, Aarhus C, Denmark; Duke University Medical Center, United States of America

Plants and pathogens evolve in response to each other. This co-evolutionary arms race is fueled by genetic variation underlying the recognition of pathogen proteins by the host and the defeat of host defenses by the pathogen. Together with new mutations, genetic diversity in populations of both the host and pathogen represent a pool of possible variants to maintain adaptation via natural selection.

Drastic changes in genetic diversity in crop species have occurred as a consequence of domestication. Whether changes in the genetic composition of these host populations also have affected genetic diversity in pathogen species is, so far, poorly understood. Advances in comparative genomics and population genomic approaches open new avenues to study adaptive processes in plant pathogens and to infer the impact of agro-ecosystems on the evolution of pathogen populations. Here we summarize new insights gained from comparative genome studies and population genomics in host-pathogen systems.

## 1. What Are the Evolutionary Consequences of Domestication in Crop Species?

In the process of domestication, crop species have undergone striking changes in both morphology and physiology. At the genetic level, these phenotypic changes have been brought by strong directional selection on a few genes [1]. Genome-wide analyses of domesticated crop species and their wild relatives have provided new insights into the evolutionary consequences of domestication. While only a modest, yet growing, number of genes associated with domestication have been identified [2]–[4], genome-wide “footprints” of domestication are well documented in several crop species [5]–[8]. Most notably the level of genetic variation has been dramatically reduced in many domesticated species relative to their wild ancestors [1]. This is best explained by intense selection on a small subset of genotypes exhibiting desirable phenotypes. Such strong directional selection also entails a number of important evolutionary side effects. First, genetic diversity can be swept away in genomic regions flanking strongly selected domestication genes [9]. Second, strong selection on one gene can reduce the efficacy of selection at neighboring loci, which, in return, may lead to an accumulation of slightly deleterious mutations. This process was first documented as an accumulation of non-synonymous mutations in Asian rice [5] and has since been termed the “cost” of domestication (see, e.g., [1], [10]). The loss of variation and the cost of domestication in genomes of crop species may compromise the level of natural defenses against pathogens and render them more susceptible than their wild relatives.

## 2. How Does the Agro-Ecosystem Affect Evolution of Plant Pathogens?

Agro-ecosystems brought by domestication have favored the emergence and specialization of new pathogen species by providing a new ecological niche. High densities of host individuals, genetic homogeneity of host populations, and recently the facilitated long distance dispersal of propagules by anthropogenic activities are factors that are greatly conducive for the propagation of pathogens in agro-ecosystems [11]. However, successful propagation in the field also poses challenges for pathogens: the new agricultural environment imposes strong directional selection on the pathogen genome, notably on genes controlling the defeat of crop resistance genes and resistance towards pesticides. Speciation in an agricultural environment can also have drastic implications for the population biology of pathogens as exemplified by the potato late blight pathogen *Phythophthora infestans* [12], the rice blast pathogen *Magnaporthe oryzae* [13], and the yellow rust pathogen *Puccinia striiformis* [14]. The emergence of these three species was associated with a strong reduction in sexual recombination and the spread of only few specialized clonal lineages. Notably, genetic diversity among “domesticated” asexual pathogen lineages was significantly reduced compared to their wild relatives. However, even when sexual recombination is maintained in the pathogen, the transition to an agricultural host can entail founder events where substantial genetic variation is lost. Comparisons of levels of genetic diversity in pathogens occurring on domesticated versus non-cultivated hosts have confirmed this hypothesis [15], [16]. Substantial loss of genetic diversity is often associated with speciation and specialization to a crop host, yet several studies report rapid emergence and adaptation of plant pathogens to crop species [17]. This states the paradox that despite relatively modest levels of genetic variation, pathogen populations can readily adapt to agro-ecosystems and rapidly respond to new introduced resistance genes, fungicides, and other disease control agents.

## 3. How Have Plant Pathogens Emerged in Agro-Ecosystems?

Comparative genomics and population genomic analyses have provided new insights into genome evolution, speciation, and the origin of pathogenicity traits. A main conclusion from these studies is that fungal pathogens can exhibit very high levels of genome plasticity and plasticity itself may be instrumental in enabling the emergence of new virulence traits [18], [19].

In the plant pathogen *Verticillium dahliae* a population genomics analysis was used to identify a determinant of race specificity [20]. Genome re-sequencing of four “race 1” isolates and six “race 2” isolates of *V. dahliae* led to the identification of a 50 kb fragment only present in race 1 isolates. Subsequent RNAseq data revealed only a single highly expressed ORF in this region. Experimental analyses confirmed the determining role of this gene as a virulence factor in race 1 strains of the pathogen. *V. dahliae* is a predominantly asexual pathogen and the acquisition of this virulence factor, possibly through a horizontal gene transfer event, underlines the importance of genome plasticity in adaptive evolution of fungal pathogens.

In addition to horizontal gene transfer, new pathogenicity traits may emerge from the crossing of genetically rather distant individuals. Population genomic analysis of the grass pathogen *Zymoseptoria pseudotritici* revealed a particular evolutionary history for this species [21]. The genome of *Z. pseudotritici* harbors a mosaic of long fragments without any polymorphisms interspersed with fragments exhibiting two distinct haplotypes. This pattern is consistent with a recent interspecific mating of just two genotypes resulting in a successful new species formation via hybrid speciation. The underlying molecular traits responsible for pathogenicity in the hybrid still remain to be identified. However, the broad distribution of *Z. pseudotritici* at its center of origin demonstrates how new genomic combinations mediated through interspecific hybridization can lead to the successful emergence of new pathogens.

## 4. How Do Fungi Adapt and Specialize to their Environment?

**Figure 1:**
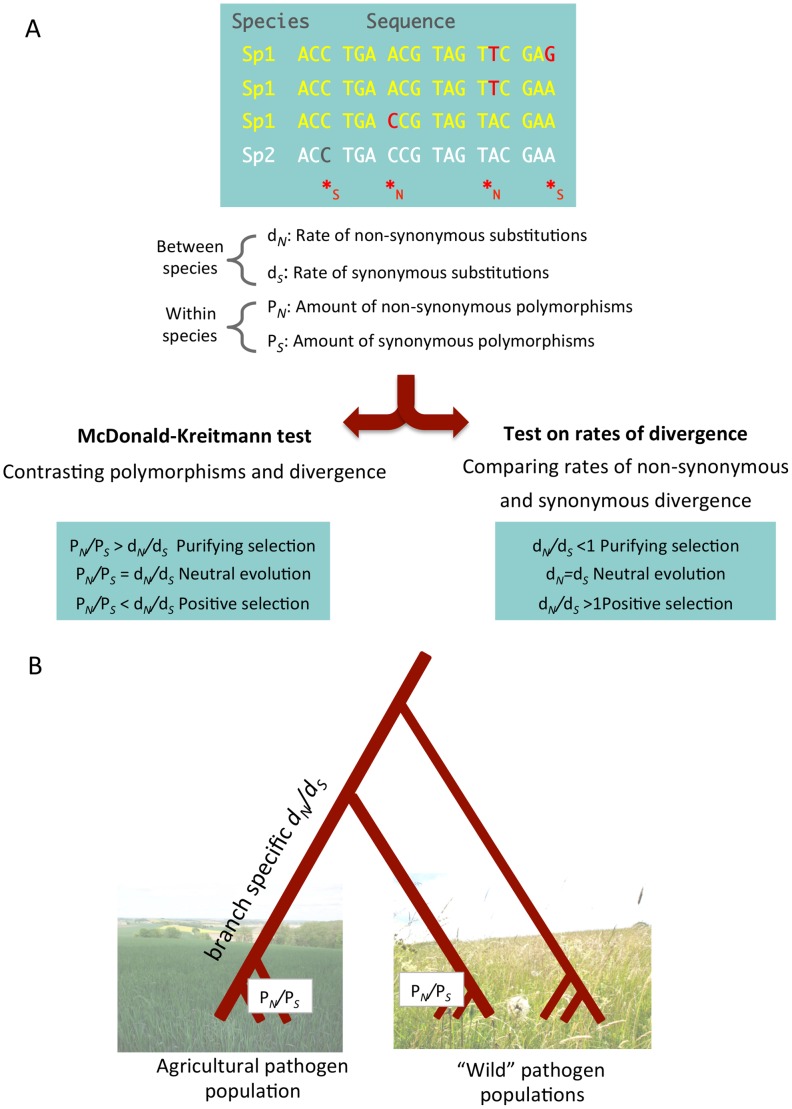
A population genomic approach allows the identification of synonymous and non-synonymous polymorphisms and substitutions. Based on these parameters, amounts of adaptive evolution and the strength of purifying selection can be quantified. (A) A multi-species (between species) and multi-genotype (within species) alignment. Nucleotide positions can be categorized as either synonymous (*S) or non-synonymous (*N). Comparison of sequences from distinct species allows the detection of sites that have undergone substitutions, while the comparison of individuals of the same species allows the detection of non-synonymous (P*_N_*) and synonymous (P*_S_*) polymorphisms. While P*_N_* and P*_S_* reflect present time nucleotide variation in a species, the two rates of divergence d*_N_* and d*_S_* inform on types of selection during the past divergence of the species. Contrasting the rates of polymorphism and divergence (using a McDonald-Kreitmann-based test [24]) provides a finer grained picture of ongoing levels of purifying selection. (B) During the divergence of a new pathogen species in an agricultural environment, non-synonymous and synonymous substitutions have accumulated in the genome. A branch-specific model [28] can infer rates of non-synonymous and synonymous substitutions as d*_N_* and d*_S_*. The d*_N_*/d*_S_* ratio provides insight into the amount of fixed substitutions since the species divergence. An increased d*_N_*/d*_S_* ratio reflects either a relaxation of purifying selection (accumulation of slightly deleterious mutations) or the fixation of adaptive mutations. To assess the strength of purifying selection we can compare ratios of non-synonymous to synonymous polymorphisms (P*_N_*/P*_S_*). A comparison of P*_N_*/P*_S_* ratios between populations can illustrate differences in evolutionary rates under different environmental conditions. Pictures are courtesy of Julien Dutheil.

Population genomics surveys allow the inference of genome-wide distributions of polymorphisms within a species and allow the detection of synonymous and non-synonymous polymorphisms in coding sequences [22]. Some important parameters can be inferred from genome-wide distributions of polymorphisms. Traces of selective sweeps can be visualized as local drops in nucleotide diversity in regions surrounding a swept locus. Such genomic patterns may lead to the identification of strongly selected alleles in a population [23]. Moreover, measures of population differentiation such as the parameter Fst can reveal loci contributing to divergent adaptive evolution among sub-populations. Deviations from a genome-wide average Fst value can reflect recent divergent selection of alleles responsible for local adaptation. The relative abundance of non-synonymous and synonymous polymorphisms (P*_N_* and P*_S_*) furthermore measures the direct effect of natural selection removing slightly deleterious non-synonymous variants in coding sequences (purifying selection). Most genes are expected to evolve under purifying selection (i.e., P*_N_*/P*_S_<1)*, however a local increase in P*_N_*/P*_S_* may reveal those few genes where new non-synonymous variants are favored by natural selection (positive selection). If genomic data from one or more related species are available, rates of non-synonymous (*d*_N_) and synonymous (*d*_S_) substitutions can be assessed to contrast within and between species patterns of variation (Figure 1). Such tests allow the detection of ancient selection in homologous genes of related species.

A number of other approaches have been developed to explore genome data and to characterize patterns of natural selection. These include methods to quantify adaptive evolution and purifying selection as well as coalescence models to infer genome evolution, recombination patterns, and effective population sizes [24], [25].

Ongoing adaptation and footprints of natural selection were reported in an elegant population genomics study of the non-pathogenic model species *Neurospora crassa.* Here the authors looked for regions exhibiting distinctly high levels of population differentiation [26] among 48 *N. crassa* isolates representing two geographically isolated populations evolving in different climatic environments. The highest Fst values between subpopulations of *N. crassa* were found in genomic islands encoding genes involved in temperature response and circadian function suggesting adaptation to different temperature and photoperiod ranges.

A population genomics approach was also used to study patterns of adaptive evolution in the wheat pathogen *Mycosphaerella graminicola* and to assess whether the specialization to an agricultural environment entailed a “domestication cost” [27]. To do so, two closely related species occurring only on hosts in natural grasslands were compared to *M. graminicola*. Patterns of non-synonymous and synonymous polymorphisms (P*_N_* and P*_S_*) were analyzed jointly with rates of branch-specific estimates of substitutions (*d_N_* and *d_S_*) to assess the effect of natural selection on coding sequences. A significant finding was that the transition to an agro-ecosystem did not entail an evolutionary “cost” in *M. graminicola*. Evolution in *M. graminicola* is instead characterized by the efficient fixation of new beneficial mutations enabling the pathogen to adapt to its new host and environment as reflected by currents ratios of P*_N_*/P*_S_* in the three species and branch-specific rates of *d*_N_ and *d*_S_. Sexual recombination in the pathogen has likely also played a significant role in the purging of deleterious mutations.

The above-mentioned studies exemplify the broad potential of population genomic analyses in evolutionary studies of plant pathogens including the identification of strongly selected genes, estimates of evolutionary potential, and inferences of past demographic histories and current levels of adaptive evolution.

## 5. What's Next: Population Genomics as a Tool in the Development of Improved Disease Management Strategies

Population genomics studies will boost our understanding of adaptive evolution of plant pathogens in agro-ecosystems. The first studies on fungal pathogens have so far shown that fungal pathogens readily adapt to the agricultural environment and have revealed strong footprints of natural selection on their genome-wide diversity. Several studies have documented unexpectedly high levels of genome plasticity in fungal pathogens, allowing the acquisition of new genetic material and the presence of supernumerary chromosomes. We still need to understand the drivers of genome plasticity and their importance for adaptation. An equally important endeavor will be to understand further how the evolutionary potential of pathogen populations is maintained in spite of strong directional selection within homogenous host environments.

In light of the increasing need to control plant pathogens, we consider the close integration of evolutionary genomics with experimental studies will be essential to describe and predict the emergence, establishment, and adaptation of plant pathogens in agro-ecosystems. A combination of evolutionary analyses of genome-wide patterns of genetic diversity in crops and pathogens combined with targeted experiments, including functional studies and experimental evolution on pathogens, will be important to guide the much-needed design of novel and sustainable strategies to slow down the emergence and spread of pathogens [10], [27].
